# Predictive and Prognostic ^18^F-Fluorocholine PET/CT Radiomics Nomogram in Patients with Castration-Resistant Prostate Cancer with Bone Metastases Treated with ^223^Ra

**DOI:** 10.3390/cancers16152695

**Published:** 2024-07-29

**Authors:** Marcos Cruz-Montijano, Mariano Amo-Salas, Javier Cassinello-Espinosa, Iciar García-Carbonero, Jose Carlos Villa-Guzman, Ana Maria Garcia-Vicente

**Affiliations:** 1 Nuclear Medicine Department, University Hospital of Toledo, 45007 Toledo, Spain; mcmontijano@sescam.jccm.es; 2 Mathematics Department, Universidad de Castilla-La Mancha, 13071 Ciudad Real, Spain; mariano.amo@uclm.es; 3 Oncology Department, University Hospital of Guadalajara, 19002 Guadalajara, Spain; jacaes@sescam.jccm.es; 4 Oncology Department, University Hospital of Toledo, 45007 Toledo, Spain; igarciac@sescam.jccm.es; 5 Oncology Department, University Hospital of Ciudad Real, 13005 Ciudad Real, Spain; jcvilla@sescam.jccm.es

**Keywords:** ^18^F-fluorocholine PET/CT, bone scintigraphy, radiomics, radium-223 dichloride, castration-resistant prostate cancer with bone metastases (CRPC-BM), therapeutic failure, overall survival, nomogram

## Abstract

**Simple Summary:**

Response to treatment and prognosis after ^223^Ra treatment varies among patients. Using a combination of clinical, biochemical, and radiomic data from bone scintigraphy and ^18^F-Fluorocholine PET/CT obtained from our prospective ChoPET-Rad study, we identified predictive and prognostic variables. The goal was to create a nomogram able to predict therapeutic failure and overall survival, aiding in the selection of more suitable candidates for this treatment.

**Abstract:**

Purpose: We aimed to develop a nomogram able to predict treatment failure, skeletal events, and overall survival (OS) in patients with castration-resistant prostate cancer with bone metastases (CRPC-BM) treated with Radium-223 dichloride (^223^Ra). Patients and Methods: Patients from the Castilla-La Mancha Spanish region were prospectively included in the ChoPET-Rad multicenter study from January 2015 to December 2022. Patients underwent baseline, interim, and end-of-treatment bone scintigraphy (BS) and ^18^F-Fluorocholine PET/CT (FCH PET/CT) scans, obtaining multiple imaging radiomics as well as clinical and biochemical variables during follow-up and studying their association with the previously defined end-points. Survival analysis was performed using the Kaplan–Meier method and Cox regression. Multivariate logistic and Cox regression models were calculated, and these models were depicted by means of nomograms. Results: Median progression-free survival (PFS) and OS were 4 and 14 months (mo), respectively. The variables that showed independent and significant association with therapeutic failure were baseline alkaline phosphatase (AP) levels (*p* = 0.022) and the characteristics of BM on the CT portion of PET/CT (*p* = 0.017). In the case of OS, the significant variables were therapeutic failure (*p* = 0.038), the number of lines received after ^223^Ra (*p* < 0.001), average SUVmax (*p* = 0.002), bone marrow infiltration in FCH PET/CT (*p* = 0.006), and interim FCH PET/CT response (*p* = 0.048). Final nomograms included these variables, showing good discrimination among the 100 patients included in our study. In the study of skeletal events, only OS showed a significant association in the multivariate analysis, resulting in an inconsistent nomogram design. Conclusions: FCH PET/CT appears to be a good tool for evaluating patients eligible for treatment with ^223^Ra, as well as for their follow-up. Thus, findings derived from it, such as the morphological characteristics of BM in the CT, bone marrow infiltration, or the response to ^223^Ra in the interim study, have proven to be solid and useful variables in the creation of nomograms for predicting therapeutic failure and OS.

## 1. Introduction

Radioisotope bone-targeted therapy can be divided into calcium analogs such as ^223^Ra and strontium-89 and bisphosphonate derivatives such as rhenium-186 etidronate. ^223^Ra, a calcium-mimetic drug, is incorporated into the bone by osteoblasts, introducing a targeted alpha therapy for the treatment of CRPC-BM in clinical practice [1,2,3,4]. 

In 2013, the ALSYMPCA study defined a significant improvement in overall survival (OS) of 3.6 months in castration-resistant prostate cancer with bone metastases (CRPC-BM), with respect to placebo, which was unique for a radiopharmaceutical therapy [5]. Following this publication, the Food and Drug Administration and European Medicines Agency (EMA) approved Radium-223 dichloride (^223^Ra) as a treatment option for symptomatic CRPC-BM patients with limited extraosseous disease. However, in 2018, ERA-223, a phase 3 randomized study promoted by the EMA, investigating the effectiveness of ^223^Ra in combination with Abiraterone in CRPC compared to a control group with placebo, reported a 29% increase in the number of fractures compared to the placebo group [6]. 

^223^Ra, as a calcium analog, is incorporated into bone by osteoblasts through the same pathway as calcium [7]. In the same manner, BS with diphosphonates spots the bone disease locations where ^223^Ra will act. However, it is well known that metastatic CRPC, as an already advanced tumor disease, promotes tumor heterogeneity, with two implications: increasing the chance of resistance to different therapies and limiting the disease detection in one-step molecular imaging, supporting the use of additional diagnostic procedures. 

The higher diagnostic accuracy of positron emission tomography/computed tomography (PET/CT) with choline analogs compared to standard BS in detecting BM, as well as the ability to diagnose extraosseous disease in the same scan, moved us to develop a prospective and multicenter study (ChoPET-Rad) using the unique PET radiotracer available and authorized for prostate cancer use in Spain, ^18^F-Fluorocholine (FCH) [8,9]. Thus, we used BS and FCH PET/CT for patient selection before ^223^Ra and treatment response based on clinical practice; monitoring ^223^Ra treatment relies on clinical and biochemical markers, while assessment of response with imaging techniques remains a controversial topic [10,11]. 

In addition, because not all patients obtain benefits from ^223^Ra, patient selection is the cornerstone of the therapy’s effectiveness, although it is a continuous challenge. In the last decade, despite efforts, predictive factors have not been established in clinical practice [12,13,14,15]. For this purpose, the development of a novel nomogram that includes clinical and imaging variables, considering findings in BS and FCH PET/CT in the prediction of therapeutic failure, could be useful for the optimal selection of those CRPC-BM patients eligible for this treatment.

Therefore, we aimed to study the value of FCH PET/CT and BS in patient selection and response assessment. Additionally, based on the scarce evidence regarding the prognostic factors potentially able to select patients most likely to benefit from ^223^Ra [16,17], a second objective was to obtain a nomogram system including clinical and radiomic variables able to predict therapeutic failure, bone events, and OS in patients with CRPC-BM who underwent ^223^Ra therapy.

## 2. Material and Methods

The present study (ChoPET-Rad) was designed as a prospective, multicenter (six centers), and non-randomized study approved by an Ethical Committee (internal code: C-52/2016). Informed consent was obtained from all patients.

### 2.1. Patients 

Patients with CRPC-BM who met all the inclusion criteria and none of the exclusion criteria for ^223^Ra treatment were included between January 2015 and December 2022. The inclusion criteria for initiating ^223^Ra treatment were (a) patients with CRPC with symptomatic BM and a negative or inconclusive CT for adenopathic involvement larger than 3 cm or visceral metastatic disease performed in the previous 6 weeks to request ^223^Ra treatment, (b) patients with a good bone marrow reserve that fulfilled the hematologic criteria necessary to administer ^223^Ra, and (c) an Eastern Cooperative Oncology Group (ECOG) performance status of 0–2 and life expectancy greater than 6 months. 

The exclusion criteria were patients (a) who declined to participate in the study, (b) who did not fulfill any of the inclusion criteria, or (c) who were diagnosed with visceral or diffuse bone marrow involvement on baseline FCH PET/CT and/or BS.

Patients were scheduled for treatment with ^223^Ra (55 KBq/kg, intravenously) in a 4-week cycle. Patients who had completed a total of six cycles of ^223^Ra were defined as treatment completion, and those who did not finish the complete treatment protocol because of clinical progression or any other cause were considered as treatment failure. Patients maintained androgen deprivation therapy. 

### 2.2. Clinical Assessment 

Each patient was clinically, hematologically, and biochemically evaluated before each ^223^Ra administration and bimonthly or monthly after the last ^223^Ra administration, depending on the patient’s clinical status and the subsequent therapeutic lines. 

Clinical variables studied were age, Gleason score, prostate-specific antigen (PSA), alkaline phosphatase (AP) and lactate dehydrogenase (LDH) levels, time of evolution of prostate cancer, time of evolution of BM, therapeutic line that ^223^Ra represented, number and type of treatments received before ^223^Ra, castration-resistance date, bone events before, during, or after ^223^Ra treatment, and having received a bone protective medication like zoledronic acid and ECOG performance status before ^223^Ra. LDH and AP were considered pathological when their values were higher than 333 and 147 U/L, respectively. Treatments received after ^223^Ra were collected.

Bone or skeletal events (SEs) were considered when BM required analgesic treatment with radiotherapy (RT) or orthopedic surgery, as well as the detection of pathological fractures or spinal cord compression syndrome, with or without the need for palliative RT.

Clinical progression was assessed following the Radiographic Assessments for Detection of Advanced Recurrence (RADAR) II group recommendation [18] when at least two of the following indicators were reached: (1) convincing and consistent rise in PSA, defined as three consecutive rises, resulting in two 50% increases over the basal PSA value, (2) diagnostic imaging progression evidence, or (3) status performance worsening or appearance of clinical symptoms while the patient was on therapy. Diagnostic imaging progression was established when any of the imaging techniques (BS or FCH PET/CT) defined compatible signs. 

^223^Ra treatment was stopped when clinical progression was addressed. However, the decision to stop treatment, based on early progression (after the third or fourth ^223^Ra doses), was made by a multidisciplinary team (oncologist and nuclear medicine physician).

Biochemical PSA response was considered when a decrease in the absolute PSA value of ≥30% between baseline PSA and interim (1 month after the third dose) or end-treatment (1 month after the sixth dose) was observed and was defined as early or delayed response, respectively. Stability was considered for the rest of the biochemical changes. AP and LDH progression was defined as an increase of ≥25% from the last available determination during treatment with respect to baseline and response as a reduction of ≥30%. The rest of the conditions were considered stable.

Clinical end-points were (a) treatment failure, defined as an incomplete ^223^Ra administration (less than six cycles) because of clinical progression of the disease or hematological toxicity and/or other clinical impairment; (b) PFS, attending to PSA evolution, defined as three consecutive rises in PSA, resulting in two ≥50% increases over the basal PSA value; and (c) OS, defined as the elapsed time between the date of the start of ^223^Ra and the date of either death or the last follow-up. The last follow-up was performed in February 2024.

Patients that received less than three doses due to bone marrow failure or constitutional syndrome earlier to assess disease progression attending to PSA values, were excluded from the response assessment group. Each cause of failure was studied and noted individually, although some of them were due to causes unrelated to the treatment.

### 2.3. Image Acquisition

FCH PET/CT and BS were performed within a time interval of 4 weeks, before the first administration (baseline), before the fourth (interim), and after the sixth (end-treatment) ^223^Ra dose. The BS was acquired 3 h after injection of 740 MBq of 99mTc-methylene diphosphonate (MDP) in three reference centers. FCH PET/CT was performed in a unique reference hospital, 5–15 min after intravenous administration of 2–4 MBq/kg, in three-dimensional acquisition mode for 3 min per bed position, from the skull to proximal legs. Low-dose CT (120 kV, 80 mA) without contrast was performed for attenuation correction and as an anatomical map. The emission data were corrected for scatter, random coincidence events, and system dead time using the provided software.

### 2.4. Imaging Evaluation

Two independent observers visually evaluated FCH PET/CT and BS. In case of discordance, a third observer reviewed the studies to reach a consensus. BM extension was assessed on baseline FCH PET/CT and BS, considering maximum intensity projection (MIP) in the former and planar images in the latter. Depending on the number of BM in BS and FCH PET/CT, BM disease was classified as oligometastatic (≤5 lesions) or polimetastatic (>5 lesions). Also, referring to the extension of the bone disease, 4 grades were established, understanding grade I as single or oligometastatic disease, grade II between 6 and 20 metastases, grade III with more than 20 metastases, and grade IV superscan pattern. When more than 4 BM were observed in BS or FCH PET/CT, with at least one extra-axial location, it was considered a high tumor burden. Furthermore, it was compared whether the predominant activity was osteogenic (BS dominant) or metabolic (PET dominant). Finally, morphological translation of BM in the CT portion of FCH PET/CT was visually assessed exclusively in pathological locations and was classified as predominantly osteoblastic (>50% of blastic lesions), osteolytic (>50% lytic lesions), or mixed (both blastic and lytic combined in a similar proportion).

The definition criteria for bone positivity on FCH PET/CT was the presence of focal tracer uptake higher than background, regardless of its intensity, with or without any underlying lesion in CT, and that could not be explained by a benign lesion like osteophytes or radiotracer excretion locations. 

Special care was taken analyzing the baseline FCH PET/CT where regions of interest were placed, obtaining the maximum standardized uptake value (SUVmax) of the most hypermetabolic BM and the average SUVmax of the five BM with the highest FCH activity. The relation between SUVmax of the hottest BM and the hepatic background was also assessed, classifying it as higher or lower than liver activity.

For lymph node evaluation, any node with visually detectable uptake (higher than background) on FCH PET/CT, despite its size, was considered suspicious of malignancy. The rest of the pelvic organs, such as the prostate, bladder, and seminal vesicles or visceral locations (lung and liver), were evaluated in the same way. Any of these were considered as soft tissue involvement (STI), except visceral metastases that caused the exclusion of the patient from receiving ^223^Ra. 

For response assessment, interim and end-treatment BS and FCH PET/CT were compared with respect to the previous one, evaluating all the included anatomical areas (preferable axial skeleton and proximal third of extremities) in order to establish response following the criteria for response formulated by the National Prostatic Cancer Treatment Group, formerly called the National Prostatic Cancer Project (NPCP) [19] and according to criteria of the European Organization for Research and Treatment of Cancer (EORTC), respectively [20]. For progression assessment, the same criteria were used for BS and FCH PET/CT, consisting of the appearance of at least 2 new lesions.

Concordance between FCH PET/CT and BS baseline, interim, and end-treatment, understanding this as a similar BM distribution between both techniques, was evaluated by visual inspection and classified as good (≥75% of the lesions), moderate (approximately between 75 and 25% of lesions), and bad (≤25% of lesions). 

## 3. Statistical Analysis 

Statistical analysis was performed using SPSS software (v. 29). Quantitative variables were represented by mean and standard deviation, and qualitative variables by frequency and percentage. The relation between qualitative variables was studied using the chi-squared Pearson test.

The Kaplan–Meier method and Cox regression were considered to study the prognostic factors of the OS and the PFS. A log-rank test was used to compare the survival curves among categories of each factor. The median follow-up was estimated using the reverse Kaplan–Meier method.

Cohen’s kappa coefficient was used to report the concordance regarding the extension of BM between BS and FCH PET/CT, classifying the results as poor (<0.20), weak (0.21–0.49), moderate (0.41–0.60), good (0.61–0.80), and very good (0.81–1.00). Multivariate analysis was carried out using logistic and Cox regression. The final models were obtained with a stepwise method. Finally, these models were depicted by means of nomograms using the package rms in R software (version 4.4.0). Statistical significance was established by a *p*-value < 0.05.

## 4. Results

Treatment with ^223^Ra was requested for 117 patients; however, after clinical and imaging data derived from BS, CT, and FCH PET/CT, 17 were dismissed for different reasons: three due to visceral metastatic disease (pulmonary, hepatic, and cerebral, respectively), three with locoregional infiltration (seminal vesicles, bladder, and pelvic lymph nodes, respectively), four due to extensive bone marrow infiltration observed by BS and/or FCH PET/CT, two with hematological toxicity (anemia and pancytopenia, respectively), one with a possible second primary tumor (hypernephroma), two for a deteriorated clinical condition with ECOG > 2, one for medullary canal infiltration visualized on FCH PET/CT, and one due to having a single BM.

Finally, 100 patients were enrolled in the current study. Clinical and disease characteristics of patients are summarized in Table 1 and Table 2. Most of them (n = 97) had a good clinical status (ECOG 0-1) before the initiation of ^223^Ra treatment with a Gleason score ≥ 8 in 45 patients. Only 53 patients underwent previous prostate cancer radical treatment: 26 had a prostatectomy (four of them received adjuvant RT due to affected surgical margins on surgical specimens), and 27 had radical RT. The remaining treatments received prior to ^223^Ra are described in Table 2. Regarding the line of treatment, ^223^Ra was administered within the first three therapeutic lines in 80 patients, so in the global list of treatments, ^223^Ra represented the third line (median). 

Forty-four patients completed six doses of ^223^Ra therapy. PSA progression was detected in 70 patients during treatment with ^223^Ra, with 54 experiencing it within the first 3 months from the start of treatment (early progression). In three patients, it was not possible to assess if there was PSA progression as they died before this parameter could be elevated.

Skeletal events before ^223^Ra initiation occurred in 23 patients and included five pathological fractures, seven spinal cord compression syndromes, three of which were treated with RT, and 11 lesions treated with RT for pain. SEs during and after ^223^Ra were documented in 3 and 26 patients, respectively, and included 15 lesions treated with RT, four pathological fractures, one of which was treated with orthopedic surgery, and seven cord compressive syndromes, three of which were treated with RT. Two cases of spinal cord compression during ^223^Ra treatment led to treatment discontinuation.

Regarding bone protective treatments, 90 patients received Denosumab, Zoledronic acid, or both during their disease management (22, 58, and 10, respectively). Forty-four patients maintained them before, during, and after ^223^Ra treatment, while the rest only maintained them in some of these circumstances.

Median follow-up was 73 months, with a median PFS and OS of 4 and 14 months, respectively. Ninety-seven patients experienced PSA progression during their follow-up; 83 patients received at least one treatment after ^223^Ra, with a mean of two subsequent lines received. Received treatments included abiraterone, enzalutamide, and one or more lines of chemotherapy in 26, 28, 41, and 28 of them, respectively. For those patients who completed ^223^Ra treatment, the median OS was 20 months compared to 9 months for those who did not complete it. During the follow-up, 92 patients died.

According to the analysis of baseline parameters of imaging techniques, only a single patient did not undergo a baseline BS prior to treatment, while 87 underwent a baseline FCH PET/CT. All patients showed a positive BS and FCH PET/CT scan. Additionally, a high tumor burden was observed in 61% of BS and 48% of FCH PET/CT. Furthermore, FCH PET/CT detected STI in sixteen patients, fourteen with nodal involvement, three with local prostatic disease, and prostatic and nodal disease in two patients. The detailed analysis of baseline parameters derived from the results of these techniques is described in Table 1 and Table 3.

If we focus on the assessment of treatment response, 13 patients were excluded for receiving less than three doses of ^223^Ra: five due to hematological intolerance, two for symptomatic bone progression, two for visceral progression, two for primarily digestive intolerance, and the last two due to other causes unrelated to ^223^Ra (traumatic fracture and pulmonary embolism).

Only seven patients experienced a PSA response during ^223^Ra treatment; four of them had early responses during the first three doses that remained during the rest of the treatment, and the rest had delayed ones during the second three doses of ^223^Ra. 

AP progression was observed in 15 cases, and LDH progression in 17. AP and LDH responses were detected in 22 and 10 cases, respectively. 

Regarding binary imaging response (progression vs. no progression), progression was more frequently observed in FCH PET/CT scans both at interim and end-treatment compared to BS. According to the progression pattern in interim FCH PET/CT, in most cases, progression was osseous, but in fifteen of them, soft tissue involvement was observed: three were exclusively nodal, seven were nodal and osseous, and five were visceral and osseous; 57% and 47% of patients progressed in interim and end-treatment FCH PET/CT, respectively. In end-treatment FCH PET/CT, the progression was eleven exclusively osseous, three nodal, one visceral, two osseous and nodal, and one osseous and visceral. The degree of agreement between interim BS and FCH PET/CT was weak (k: 0.349; *p* < 0.001), being higher in the case of binary response assessment (progression vs. no progression) (k = 0.447; *p* < 0.001). No agreement was observed between end-of-treatment studies (k = 0.157; *p* = 0.120 and k = 0.211; *p* = 0.075) in binary response. The distribution of results is shown in Table 4 and Table 5.

The results of the chi-square analysis between the different variables and therapeutic failure rate and SEs during or post ^223^Ra are summarized in Table 6 and Table 7. The number of patients who did not complete treatment with ^223^Ra (therapeutic failure) was associated with those with poorer performance status (ECOG > 0) (*p* = 0.038), those who did not receive prior bone protective treatment (*p* = 0.040), or with pathological baseline AP levels (*p* = 0.030) or LDH (*p* = 0.039), among other factors. 

In the case of SEs, only variables as additional treatments after ^223^Ra, OS (≤12 months vs. >12 months), and a high tumor burden on FCH PET/CT showed significant associations (Table 7). Paradoxically, patients with a higher number of lines of treatment following ^223^Ra showed a statistically significant occurrence of new SEs during or after ^223^Ra treatment (*p* < 0.001). In fact, any additional therapeutic line increased the risk by 42.7%. However, in multivariate analysis, only OS showed a significant association with SEs, as patients with OS longer than 12 months had 10.795 times increased risk of SEs (*p* = 0.003). However, we interpreted this result as inconsistent for a nomogram design based on the higher the OS, the higher the probability of receiving subsequent therapeutic lines and suffering SEs.

**Figure 1:**
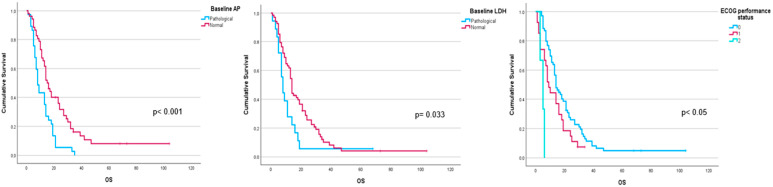
Kaplan–Meier OS curves of baseline AP levels (**upper left panel**), LDH levels (**upper right panel**), and ECOG performance status (**lower panel**).

**Figure 2:**
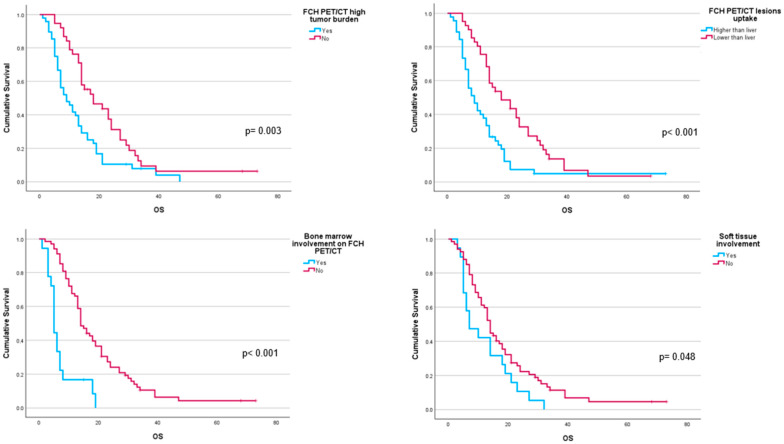
Kaplan–Meier OS curves of baseline FCH PET/CT radiomics: high tumor burden (**upper left panel**), uptake higher than liver for the most hypermetabolic bone metastases (**upper right panel**), bone marrow involvement (**lower left panel**), and soft tissue involvement (**lower right panel**).

**Figure 3:**
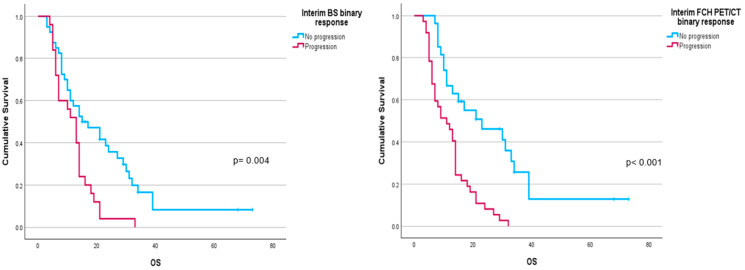
Kaplan–Meier OS curves of binary response in interim FCH PET/CT scans (**left panel**) and BS (**right panel**).

Performance status before ^223^Ra (*p* < 0.001), AP baseline levels (*p* < 0.001), and LDH (*p* = 0.033) were found to have a significant impact on OS (Figure 1), as well as other factors such as completion of treatment with ^223^Ra (*p* < 0.001). Regarding imaging variables, both the chi-square and the log-rank tests showed that derived FCH PET/CT variables had statistical significance. Thus, high tumor burden (*p* = 0.003), uptake of the most hypermetabolic lesion above the liver (*p* < 0.001), or STI in the FCH PET/CT study (*p* = 0.048) were associated with a lower median OS (Figure 2). Focusing on treatment response assessment, only interim studies (BS and FCH PET/CT) showed a significant association with OS, with data extracted from FCH PET/CT being slightly more robust compared to BS (*p* < 0.001 vs. *p* = 0.004, respectively) (Figure 3). The significant variables in the univariate analysis were included in the multivariate analysis for the OS and therapeutic failure end-points. In the former, the association of uptake of BM above the liver (*p* = 0.011), therapeutic failure (*p* = 0.001), or pathological baseline AP (*p* = 0.011) was highlighted as leading to lower OS. Table 8 details the results of the univariate Cox regression, and Table 9, Table 10 and Table 11 show the results of the log-rank test regarding OS.

**Figure 4:**
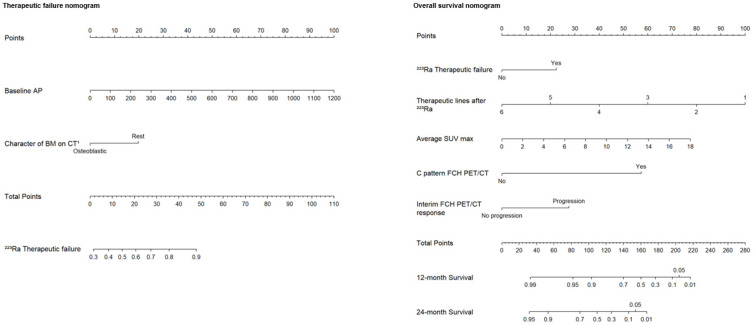
Designed nomograms to predict therapeutic failure (**left panel**) and the probability of survival at 12 and 24 months (**right panel**). The nomograms were developed based on the 100 patients in our population with CRPC-BM who received ^223^Ra therapy. To obtain the probability of therapeutic failure and survival, the values for each variable of the patients included in each nomogram are marked. Then, a straight vertical line is drawn up to the “Points” line at the top of the nomogram. This determines how many points are attributed to each variable. Once this is performed for each variable, the sum of all the points obtained is calculated and added to the “Total Points” line at the bottom of the nomogram. This value is then used to assess the individual probability of predicting the risk of therapeutic failure (**left panel**) and survival at 12 and 24 months (**right panel**).

**Figure 5:**
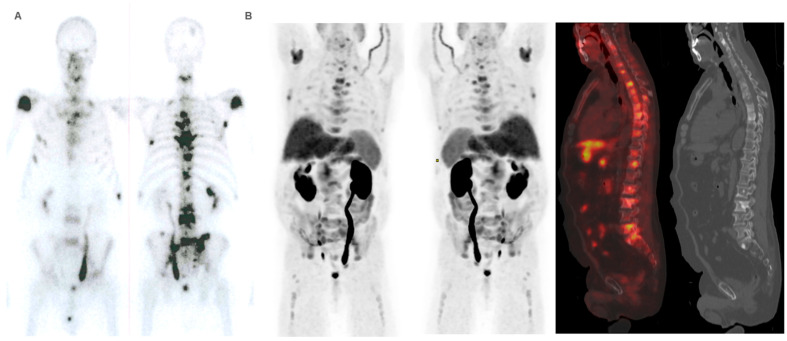
Patient ≠ 1. A 71-year-old man, diagnosed with prostate adenocarcinoma Gleason Score 9 (4 + 5), underwent prostatectomy followed by adjuvant radiotherapy to the surgical bed due to early PSA progression. Three years after diagnosis, bone metastases were detected, prompting the start of systemic treatment lines (Enzalutamide and Docetaxel). ^223^Ra was administered as the third line. At the start of treatment, the patient was in good general condition (ECOG 0), experienced pain, and had baseline PSA levels of 50.7 ng/dL, AP of 377 IU/L, and LDH of 377 IU/L. Baseline BS (**A**) shows polymetastatic disease (>20 lesions) affecting both the axial and extra-axial skeleton. Baseline FCH PET/CT (**B**) shows the presence of mixed characteristic BM, bone marrow infiltration, and uptake of the most hypermetabolic BM higher than liver and soft tissue involvement at the pelvic lymph nodes. The concordance between both studies was moderate, defining FCH PET/CT more BM with respect to BS.

**Figure 6:**
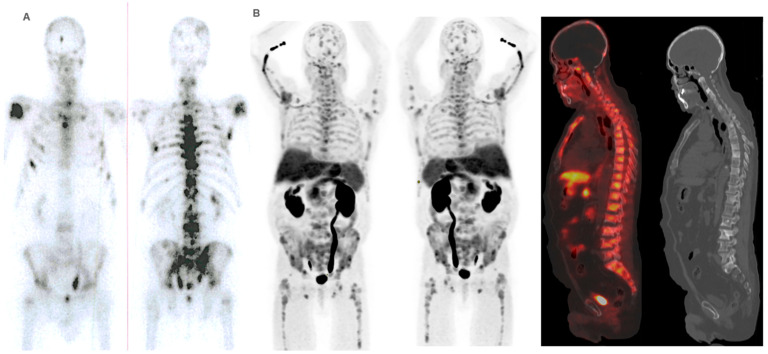
Patient ≠ 1. PSA and AP showed a steady increase after ^223^Ra initiation. Clinical deterioration was observed after the third ^223^Ra administration. Interim BS (**A**) and FCH PET/CT (**B**) show disease progression. The patient died 6 months after starting treatment with ^223^Ra.

**Figure 7:**
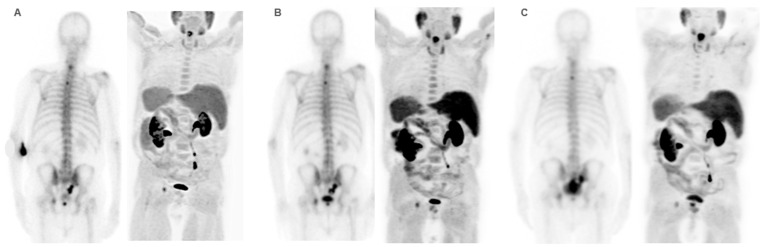
Patient ≠ 2. An 88-year-old man diagnosed with metastatic prostate cancer, Gleason Score 7 (4 + 3), from the onset. He received first-line treatment with Enzalutamide until biochemical and bone progression after 5 years. Treatment with ^223^Ra was proposed as second-line. Baseline BS and FCH PET/CT (**A**) show oligometastatic disease with only axial involvement and predominantly osteoblastic. At the start of ^223^Ra treatment, the patient was in very good general condition (ECOG 0) with baseline PSA levels of 14.9 ng/mL, AP of 73 IU/L, and LDH of 450 IU/L. The patient completed 6 doses of ^223^Ra, remaining stable in the interim BS and FCH PET/CT studies (**B**) but showed bone progression at the end of treatment in both the BS and FCH PET/CT (**C**). His PSA levels increased during the treatment, while AP levels remained stable. After ^223^Ra treatment, the patient received Docetaxel and experienced a bone event that consisted of a painful metastatic bone at 17 months, treated with palliative vertebral radiotherapy. He ultimately died with an OS of 30 months.

**Figure 8:**
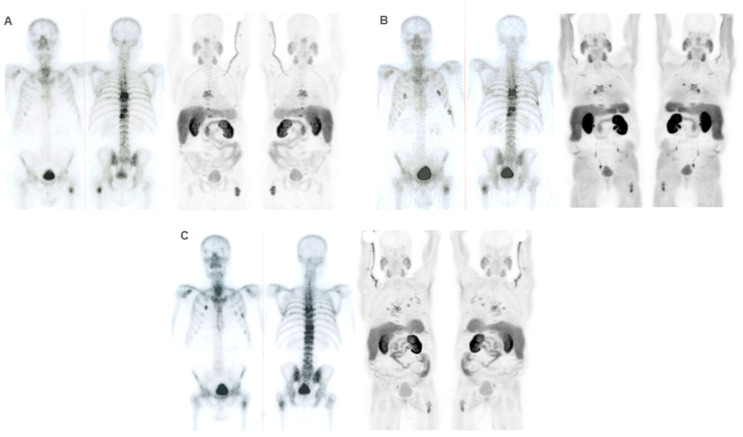
Patient ≠ 3. A 69-year-old man diagnosed with prostate cancer, Gleason score 8 (4 + 4) stage IV T3N1M1 (hepatic, pulmonary, nodal, and bone infiltration). The patient achieved a complete radiologic response after receiving first-line Docetaxel with an anti-androgen blockade. After progression, Abiraterone–Prednisone was administered with a partial biochemical response. ^223^Ra was administered as the third line. Baseline BS and FCH PET/CT (**A**) show oligometastatic axial and extra-axial BM on the BS and polymetastatic (6–20 lesions) axial and extra-axial involvement on the FCH PET/CT without soft tissue involvement, with some BM showing lytic characteristics and higher uptake than the liver. Despite this, the concordance between both techniques was good, with metabolic activity predominating over osteogenic. At the start of treatment, the patient was in very good general condition (ECOG 0) with pain and baseline PSA values of 5 ng/dL, AP of 87 IU/L, and LDH of 363 IU/L. He received 6 doses of ^223^Ra, with PSA values progressing from the start of treatment, although AP and LDH values did not. The interim BS study shows rib deposits interpreted as bone progression while the FCH PET/CT showed stability (**B**)**,** explained by the interpretation of rib deposits as probable fractures. However, the end-treatment scans (**C**) show bone and nodal progression of FCH PET/CT with stability in the BS. Subsequently, treatment with Abiraterone–Prednisone was resumed, followed shortly after by Cabazitaxel. The patient died 10 months after ^223^Ra initiation.

Based on the independent risk factors obtained from multivariate logistic regression analyses, two nomograms were constructed: one to predict the percentage of therapeutic failure and the other to predict the 1- and 2-year survival rates in patients treated with ^223^Ra. A point scale from 0 to 100 was used to score each variable, and then the sum of all scores was calculated. Therefore, the risk of therapeutic failure and death could be predicted by observing the total points. In the first therapeutic failure nomogram, the variable characteristics of BM on the CT were divided into osteoblastic or rest of lesions (lytic or mixed), and the units of measurement for baseline AP were IU/L. In the OS nomogram, the variable lines of treatment after ^223^Ra were divided into six to one line and for the mean SUVmax from 0 to 18. In the case of the response variable on the FCH PET/CT, it was divided into progression and non-progression, and for the rest of the variables, no and yes (Figure 4). Figure 5, Figure 6, Figure 7 and Figure 8 show some representative cases.

## 5. Discussion

### 5.1. Impact on Treatment Response and OS of ^223^Ra Therapeutic Line

After the ERA-223 trial, the EMA went further, not only limiting itself to the combination of ^223^Ra with androgen receptor-targeted agents but also to its prescription as monotherapy, restricting its use, inexplicably, to those patients with CRPC with ≥6 BM who had received at least two systemic therapies administered before ^223^Ra [21]. Following this new indication, the reported OS defined by ^223^Ra use has decreased with respect to the defined OS in ALSYMPCA or previous works, with median OS ranging from 6 to 12.8 months [22,23,24,25,26,27]. The delay of patient’s inclusion for ^223^Ra treatment can be the explanation, allowing cancer cells to adapt to the selective pressures imposed by previous therapies and facilitating the development of a new resistant phenotype, making it less sensitive to obtaining the benefit of subsequent treatments in terms of response and, most important, OS [26,28,29]. 

In the present work, despite the delayed use of ^223^Ra, our median OS (14 months) was in line with ALSYMPCA (14.9 months). However, previous works defined higher median OS (around 17 months), probably explained by the patient’s characteristics, as a less castration resistance evolution time, lower values of AP, and a reduced number of previous systemic treatments [30,31,32]. 

### 5.2. Diagnostic Imaging in Patient Selection 

Patients’ benefits and outcomes after ^223^Ra treatment are strongly influenced by patient selection [5,6,7,8,9,10,11,12,13,14,15,16,17,18,19,20,21,22,23,24,25,26,27,28,29,30,31,32,33]. Some biomarkers have been suggested that may benefit from treatment with ^223^Ra and for monitoring, such as patient characteristics, findings in various imaging techniques, serum levels of collagen metabolism products, soluble factors secreted by osteoblasts, and even molecular aberrations, although with limited evidence [34,35]. 

Based on patients with shorter OS, mostly in the later stages of the disease, novel imaging techniques such as PET/CT could partially solve the suboptimal selection of patients attending to clinical criteria after EMA restrictions [26]. However, current guidelines, such as those from the European Association of Urology and the American Urological Association, do not include recommendations about diagnostic imaging assessment in patient selection and treatment monitoring of ^223^Ra in clinical practice [1,2,3,4]. In fact, BM are not typically considered in response evaluation criteria in solid tumors (RECIST) on CT in clinical trials, and BS has a limited accuracy [36,37]. Consequently, baseline-imaging parameters in CRPC-BM, which may predict response to ^223^Ra therapy, are desirable.

Choline analogs–PET/CT and other PET radiopharmaceuticals, such as ^68^Ga or ^18^F- prostate-specific membrane antigen (PSMA) and ^18^F-NaF, have shown promising results in the assessment of CRPC, so they could also be a good option for patient selection and response evaluation [38,39,40]. However, imaging restrictions and under-recognition have promoted that the role of these novel imaging techniques, based on PET/CT, has not been well documented. Recent work revealed that baseline PSMA PET/CT versus CT did not seem to impact biochemical response during ^223^Ra therapy in mCRPC patients. Nevertheless, patients in the baseline CT group had a significantly shorter OS compared to the PSMA PET/CT group (12.4 vs. 19.9 months, respectively), most likely due to under-detection of STI in the former. Therefore, replacing CT with PSMA PET/CT appears to be a valuable screening method for identifying patients who will benefit most from ^223^Ra therapy [41]. 

PET/CT-derived functional parameters, such as maximum and mean SUV and lean body mass corrected SUV peak (SUL peak), have been investigated as potential biomarkers for patients’ prognostication before therapy. Previous works using baseline FCH PET/CT have shown controversial results, with no associations of SUVmax with response to treatment [39,40,41,42] and a negative correlation of SUVmax with PFS and OS in patients receiving enzalutamide [43,44]. Furthermore, volumetric SUV-based parameters, namely metabolically active tumor volume (MATV) and total lesion activity (TLA), both reflecting the burden of metabolically active disease, were found to have a significant prognostic impact [42,44,45,46]. In addition, FCH PET may be useful for patients’ stratification before ^223^Ra therapy, with high MATV the only reported significant predictor of OS (*p* = 0.003; HR = 7.6) in multivariate Cox analysis [42]. In the current study, both the SUVmax value of the lesion with the highest uptake and the average SUVmax of the five lesions with the highest uptake were evaluated in the FCH PET/CT studies, showing both a significant association with OS. Additionally, perhaps a more robust or comparable among works variable, such as the existence of at least a lesion with uptake higher than the liver, proved to be another independent variable associated with OS (Figure 5 and Figure 6).

Given the similar uptake properties of ^223^Ra and BS tracer in osteoblastic bone, a reasonable hypothesis is that metastatic disease with higher uptake on BS or ^18^F-NaF PET/CT compared to FCH PET/CT, is more likely to respond to ^223^Ra therapy, based on that increased choline levels can reflect on more aggressive tumors [39,40,41,42,43,44,47,48]. Ahmadzadehfar et al. [49] reported that when PSMA PET/CT is used as the gatekeeper in addition to BS, radionuclide therapy with ^223^Ra might be more effective and have more success regarding changes in the PSA, mainly because of better patient selection with the exclusion of patients with bone marrow involvement or STI. In our case, similar results were found with the combined use of FCH PET/CT and BS, as some of the FCH PET/CT variables had significant associations with OS. These included bone marrow infiltration (median 6 vs. 14 months; *p* < 0.001), uptake of the most hypermetabolic BM above the liver (median 10.5 vs. 16 months; *p* < 0.001), and STI (median 7 vs. 14 months; *p* = 0.048).

### 5.3. Response Assessment

Disease progression on imaging techniques is the most critical parameter for therapeutic change in mCRPC. Besides the limited value of CT and BS for early response evaluation of BM, blood-based biomarkers, including PSA and AP, have shown controversial results. Serum AP has been defined as a very useful marker to assess ^223^Ra treatment response by some authors [16]. However, in clinical practice, an AP decrease after ^223^Ra treatment has not been necessarily associated with tumor response on ^68^Ga-PSMA PET/CT and could coexist with radiologic disease [38]. In fact, AP reflects osteoblasts activity, which is targeted by ^223^Ra, but does not directly reflect the tumor load. On the other hand, in several studies, a decline in AP levels during ^223^Ra therapy has been associated with improved OS [5,12,50]. Van der Doelen et al. [51] observed that those patients with elevated baseline AP who did not show a ≥10% reduction after the first dose of ^223^Ra had worse OS compared to those who did reduce their baseline AP values, which could be an early indicator of treatment resistance. In our work, we did not study this association mainly because few patients experienced a reduction of these biochemical markers during ^223^Ra treatment. 

Regarding BS, BSI has been defined as very useful for both evaluating treatment response and predicting the prognosis of treated patients [16]. In the present work, BM progression was established on BS using NPCP criteria, although without the needed confirmation on a second scan according to the 2 × 2 rule. In addition, for a more reproducible comparison, we used the same criteria for FCH PET/CT. Only interim BS and FCH PET/CT showed significant association with OS with the strongest relation of binary response (progression vs. no progression) for interim FCH PET/CT (Table 10). 

The Prostate Cancer Working Group pays little attention to PSA changes during and after therapy cycles. However, using PSMA PET/CT as a supportive imaging diagnostic technique for response evaluation, there was a significant correlation between PSA changes and the treatment response according to images, which increases the value of PSA as a reliable tumor marker for the follow-up of these patients [49]. According to our data, we completely agree with the previous assessment, observing that a significant increase in the PSA level during therapy cycles likely occurred because of disease progression on FCH PET/CT. In fact, PSA change has been defined as a very useful marker for prognosis prediction [16]. In the present work, PSA progression was associated with a lower OS (median 12 vs. 19 months; *p* = 0.004). Significant results were also observed when evaluating by periods, comparing progression during the first three doses of ^223^Ra vs. any other time (median 12 vs. 16 months, respectively; *p* = 0.017). 

De Jong et al. [38] compared ^68^Ga-PSMA PET/CT with conventional modalities for response evaluation after ^223^Ra treatment in patients with mCRPC. After three cycles of ^223^Ra treatment, BS could not distinguish good responders from poor responders. At the end of treatment and at treatment failure, all patients had progressive disease on ^68^Ga-PSMA PET/CT due to the development of at least one new BM, whereas 15% and 43% of patients, respectively, also had progressive disease on conventional imaging. In addition, the interpatient heterogeneity in response was not reflected by PET Response Criteria in Solid Tumors (PERCIST) criteria. Therefore, PERCIST was not considered sufficient to distinguish good responders from poor responders to ^223^Ra treatment, and the authors decided to assess novel parameters of ^68^Ga-PSMA PET/CT as total tumor volume and voxel-wise heterogeneity. 

Summarizing, standardizing, and actualizing PET/CT response criteria is necessary, and whereas no diagnostic response criteria for FCH PET/CT exist, several criteria have been described for PSMA ligands PET/CT [52,53,54]. We used the same progression criteria for BS and FCH PET/CT in accordance with PSMA PET progression criteria defined by Fanti et al. [53]. However, perhaps a combined assessment of tumor volume variations should define a more comprehensive response definition. 

### 5.4. Skeletal Events

Although the most reported symptomatic SEs in CRPC-BM are the use of external-beam radiotherapy and symptomatic pathologic fractures, the latter, causing in some patients cord compression, are major contributors to morbidity and mortality [41,55].

The PARABO study revealed that the number of SEs decreased in patients who completed the six doses of ^223^Ra [31]. In addition, in the ALSYMPCA study [5], ^223^Ra prolonged the time to the first SE versus placebo (median of 15.6 vs. 9.8 months, respectively) when each was used in combination with the standard of care. In our population, there was no significant decrease in the number of SEs in those patients who completed ^223^Ra treatment with respect to those who had a therapeutic failure (25% vs. 27.3%, respectively). However, we observed a delay in the occurrence of these SEs, with a median of 19 months compared to 9 months, respectively. In our sample of patients, no significant association was found between SEs during or after ^223^Ra treatment and therapeutic failure (Table 6), and although SEs were a reason for ^223^Ra discontinuation in three cases, most of the SEs occurred after ^223^Ra treatment. 

Moreover, our rate of SEs during ^223^Ra treatment was lower than the previously reported 15% by Palmedo et al. [31], which additionally defined no clear impact of bone health agents on SEs in similarity with our results. However, we found a significant association between SEs during and after treatment with ^223^Ra and the number of subsequent lines of treatment received, as well as the OS of these patients. These findings suggest that the presence of subsequent SEs increases in parallel with the survival of these patients as an inherent secondary effect of BM and bone metabolism disturbances due to age and androgen deprivation (Figure 7); in fact, any additional therapeutic line increased the risk by 42.7%. However, in multivariate analysis, only OS showed a significant association with SEs, as patients with OS longer than 12 months had 10.795 times increased risk of SEs. Additionally, a relationship was found between SEs, high tumor burden, and BM involvement in the FCH PET/CT but not in the BS, which could potentially be an important factor for selecting which patients could benefit from closer monitoring. The incidence of SEs in the present work (26%) was in accordance with respect to the 33% reported by the ALSYMPCA trial [56,57] and also by Bosch et al. [41] during and after ^223^Ra therapy (36.4% and 28.8% of patients selected with baseline-PSMA vs. baseline-CT, respectively), the latter possibly in the context of the presence of a higher volume of bone disease in the PSMA group (66.7% vs. 48.1%) or a shorter OS of the CT group.

### 5.5. Therapeutic Failure

Therapeutic failure, which is understood as not completing treatment with ^223^Ra, is variable among studies ranging from 20 to 45% [41,42,58]. Previous reports indicate that patients with good prognostic factors are more likely to complete six cycles of ^223^Ra therapy [14,58,59,60]. Of those, Alva et al. [14] demonstrated that treatment with the full six cycles of ^223^Ra therapy was associated with an ECOG performance status of 0–1, no or mild pain, lower PSA, normal AP, no prior Abiraterone–Enzalutamide therapy, and low BSI, a parameter of bone tumor burden. 

In our population, it was similarly observed that a better ECOG performance status, normal baseline AP levels, and a lower number of lesions on BS were associated with completing treatment with ^223^Ra. Additionally, other variables such as having received a prostatectomy as the initial radical treatment, normal baseline LDH levels, no PSA progression during treatment, and receiving bone protective treatment were significantly associated. In multivariate analysis, the association of therapeutic failure with the absence of PSA progression during treatment was likely related to the fact that PSA progression was one of the criteria for discontinuing the ^223^Ra treatment.

Multiple baseline radiomic variables derived from FCH PET/CT, contrary to BS, showed significant associations with therapeutic failure as location, number, and extent of BM, activity higher than liver uptake, and BM characteristics on the CT portion of PET/CT (Table 6).

There was not only a significant association, as with BS, between a higher number of BM and therapeutic failure, but also with others such as axial and extra-axial BM location, having more than 20 lesions or a superscan pattern, the presence of lytic or mixed BM, STI and BM uptake higher than liver uptake. These findings suggest that FCH PET/CT could aid in selecting the best candidates for receiving ^223^Ra treatment (Table 6).

### 5.6. Prognostic Factors

Various reports have indicated several prognostic indicators associated with good OS for CRPC-BM patients treated with ^223^Ra, though the details presented differ, and no single method has yet been established. Among factors associated with a better OS are ECOG performance status of 0 [25,33,51,61], a normal value of AP [33,51,62], a lower BSI [15,51,63,64], a lower number of BM (<20) in BS [30], a normal LDH [15,27], lower PSA values [65], or higher PSA duplication times before ^223^Ra treatment [30,61].

Although a substantial survival benefit has been described in patients reaching the completion of ^223^Ra cycles [66,67,68,69,70], a controversial theory is that the ^223^Ra effective dose is only achieved by giving all six cycles and that anything less is under-treatment [71]. In the present work, therapeutic failure was a subrogate of disease progression that supports the assessment that the most likely cause of the shorter OS in patients receiving incomplete ^223^Ra treatment is the early progression caused by an advanced stage of their disease. 

Extraosseous involvement or STI on PSMA PET, defined as visceral or lymph node metastases after therapy, has been previously associated with significantly shorter OS compared with those without newly detected STI (median OS, 10.6 vs. 14.9 months, respectively; *p* < 0.01) [41]. In our study, however, no differences in OS were detected between both groups (median of 19 months vs. 17 months, respectively). Significant differences were only found in baseline studies comparing patients with STI to those with preserved soft tissues, with a median OS of 7 months vs. 14 months, respectively. 

Regarding the response to ^223^Ra treatment, our results showed a significant association between non-progression in both BS and FCH PET/CT in interim studies and greater OS, being more notable with FCH PET/CT (Table 11 and Figure 3). 

With respect to the impact derived from the association of different prognostic factors, Bauckneht et al. [26] referred to a reliable prognostic scoring system not only before the EMA but also after the EMA restriction. They obtained the referred score using four parameters: neutrophil-to-lymphocyte ratio (< vs. ≥3.1), ECOG performance status (0–1 vs. 2–3), number of BM (< vs. ≥6) and AP (< vs. ≥220), and identifying three distinctive prognostic groups (low-risk, intermediate-risk, and high-risk). In the Kitahima et al. [58] nomogram, unfavorable prognosis in patients treated with ^223^Ra after the EMA amendment was based on some characteristics such as pre-treatment with chemotherapy, lymphadenopathies, high BM tumor burden (depicted by the number of BM at the BS and PSA levels), and low hemoglobin levels. Notably, a more advanced disease was associated with lower OS. Novel nomograms developed in the present study, using FCH PET/CT, to predict therapeutic failure and prognosis following ^223^Ra treatment seem relevant for the selection of eligible patients who would receive the greatest benefit. In the therapeutic failure nomogram, baseline AP and character of BM on CT (osteoblastic vs. the rest) were robust predictive variables. So, a non-predominant osteogenic BM and high values of AP were associated with poorer response and treatment discontinuation due to progressive disease. This result is well supported by the biological mechanism of ^223^Ra and the direct association of osteoblastic disease and AP levels. Thus, these results highlight the importance of an integral evaluation of PET-positive BM and their correspondence on CT [72,73].

On the other hand, in the OS nomogram, several variables after multiple readjustments could be incorporated, such as therapeutic failure, number of therapeutic lines after ^223^Ra treatment, average SUVmax, bone marrow involvement on baseline FCH PET/CT (C pattern), and categorical interim response on FCH PET/CT (progression vs. no progression). SEs during and after ^223^Ra treatment, contrary to ERA-derived publication [6], were associated with an increase in OS in our population, possibly because a longer patient survival conditions their appearance in patients with damaged bone health based on bone destruction by metastatic infiltration added to secondary osteoporosis. However, we extracted this variable from the nomogram, considering that SEs are a consequence of survival but not the contrary. Similarly, the higher the patient’s survival, the higher the probability of receiving additional therapeutic lines. Lastly, the inclusion of FCH PET/CT as the only significant imaging variable in the nomogram highlights the potential importance of this technique in patients who underwent ^223^Ra treatment.

This study has some limitations, such as the fact that not all patients were evaluated with both imaging techniques and the limited sample of response scans, especially end-treatment studies, mainly explained by the high rate of progression and therapeutic failure. Additionally, although the same experts assessed the studies, some scans were acquired using different equipment, which could limit more reliable comparisons. In BS, only planar images were obtained in most of the patients, which restricted an effective comparison with a pure tomographic technique such as PET/CT. However, to partially palliate this limitation, only MIP images were considered for BM classification on FCH PET/CT. Moreover, no standardization exists regarding the therapeutic management after ^223^Ra treatment. This heterogeneity could affect the OS results. Lastly, no external validation of this nomogram has been performed.

Under the current EMA guidance, patients are offered ^223^Ra at a later stage in their treatment pathway. The current personalizing medicine demands the creation of validated and simple models for clinicians to assess, which patients will most likely benefit from ^223^Ra treatment. This justifies the need to find advanced and novel tools that allow for patient selection and earlier evaluation of response or progression to treatment. The hypothesis that the higher diagnostic accuracy of PET/CT radiotracers, such as FCH, could lead to better patient selection and, therefore, impact patient outcomes of the proposed treatment is the cornerstone of the present investigation. In addition, this study reveals that SEs seem not to have a detrimental influence on patient survival.

## 6. Conclusions

The present work aimed to establish a nomogram, adding FCH PET/CT to different clinical variables, to determine which patients would obtain the maximum benefit from ^223^Ra treatment, reducing as much as possible therapeutic failure and early death. Baseline FCH PET/CT is offered as a more robust predictive and prognostic imaging variable compared to BS, whereas baseline AP and a non-dominant osteoblastic pattern in CT of PET/CT seem to have relevance in the prediction of ^223^Ra treatment discontinuation. Interestingly, therapy-related variables and baseline and interim FCH PET/CT played a role in defining OS in our sample of patients. Thus, FCH PET/CT, irrespective of current conventional imaging techniques, could guide clinical decision-making on the continuation of ^223^Ra treatment in patients with CRPC with BM, although the results need to be discussed and validated in future works based on new imaging tools.

## Figures and Tables

**Table 1:** Patient and disease characteristics.

Baseline Quantitative Variables	Mean ± IQR
Age (years)	72.72 ± 8.50
Baseline PSA (ng/mL)	188.53 ± 913.94
Baseline AP (U/L)	176.78 ± 187.26
Baseline LDH (U/L)	400.46 ± 192.37
SUVmax	8.62 ± 5.37
Average SUVmax	6.82 ± 4.01
Time variables	Median (Q1–Q3)
Time of evolution of prostate cancer (months)	62.50 (75.75–108.00)
Time of evolution of bone metastases (months)	28.00 (20.00–48.00)
Time of castration resistance (months)	21 (12.25–43.00)

SD: standard deviation, AP: alkaline phosphatase, LDH: lactate dehydrogenase, PSA: prostate-specific antigen, IQR: interquartile range.

**Table 2:** Patients and disease qualitative and categorical characteristics (n = 100).

Clinical Characteristics	n and %
Gleason	
≤7	46
≥8	45
n.a.	9
Baseline AP	
Pathological	37
Normal	53
n.a.	10
Baseline LDH	
Pathological	18
Normal	69
n.a.	13
Bone events before ^223^Ra	
Yes	23
No	77
Bone events during or after ^223^Ra	
Yes	26
No	74
Bone protective treatment	
Yes	90
No	10
Death (*)	
Yes	92
No	8
Progression (*)	
Yes	97
No	3
Therapeutic failure	
Yes	56
No	44
ECOG performance status	
0	70
1	27
2	3
Enzalutamide before ^223^Ra	
Yes	42
No	58
Abiraterone before ^223^Ra	
Yes	66
No	34
ChT	
First-line Docetaxel	71
Second-line ChT	21
Xofigo line	
1	3
2	20
3	57
4	13
5	6
7	1
PSA progression during ^223^Ra	
Yes	70
No	27
n.a.	3
PSA progression during ^223^Ra (detailed 1)	
First 3 months	54
4–6 dose	16
n.a.	30
PSA progression during ^223^Ra (detailed 2)	
First 3 months	54
Rest	43
n.a.	3

n: number, %: percentage, AP: alkaline phosphatase, LDH: lactate dehydrogenase, n = number of patients, BM: bone metastases, BS: bone scan, (*) during follow-up, ChT: Chemotherapy, n.a.: not available, ECOG: Eastern Cooperative Oncology Group.

**Table 3:** Results of baseline imaging techniques (n = 100).

Baseline Imaging Characteristics	n and %
Lesion localization on BS	
Axial	39
Axial and other	60
n.a.	1
Number of BM on BS	
Oligomts £5	27
Polimts > 5	72
n.a.	1
Extent of BM on BS	
1–5 lesions	27
6–20 lesions	35
>20 lesions	30
Superscan	7
n.a.	1
High tumor burden on BS	
Yes	61
No	38
n.a.	1
Lesion localization on FCH PET/CT	
Axial	39
Axial and other	48
n.a.	13
Number of BM on FCH PET/CT	
Oligomts £5	27
Polimts >5	60
n.a.	13
Extent of BM on FCH PET/CT	
1–5 lesions	27
6–20 lesions	33
>20 lesions	21
Superscan	6
n.a.	13
High tumor burden on FCH PET/CT	
Yes	48
No	39
n.a.	13
BM characteristics on CT (*)	
Osteoblastic	56
Osteolytic	12
Mixed	19
n.a.	13
Soft tissue involvement on FCH PET/CT	
Yes	19
No	68
n.a.	13
Concordance BS/FCH PET/CT	
Good	58
Moderate	12
Bad	16
n.a.	14
A pattern on BS/FCH PET/CT	
1 (predominant metabolic activity)	22
2 (predominant osteogenic activity)	54
3 (similar uptake in both)	10
n.a.	14
B pattern (bone marrow involvement on BS)	
Yes	23
No	76
n.a.	1
C pattern (bone marrow involvement on FCH PET/CT)	
Yes	18
No	69
n.a.	13
D pattern (FCH PET/CT uptake)	
1 (higher than liver)	45
2 (lower than liver)	42
n.a.	13

n: number, %: percentage, BS: bone scan, BM: bone metastases, FCH PET/CT: 18F-fluorocholine positron emission tomography/computed tomography, (*) CT portion of FCH PET/CT, n.a.: not available.

**Table 4:** Response results of interim bone scan and FCH PET/CT.

	Interim FCH PET/CT			
		PR	S	P
Interim BS (n = 63)	PR	1	0	1
	S	7	16	14
	P	1	2	21

**Table 5:** Response results of end-treatment bone scan and FCH PET/CT.

	End-Treatment FCH PET/CT				
		CR	PR	E	P
End-treatment BS (n = 38)	PR	1	0	2	1
	E	1	1	14	12
	P	0	0	1	5

CR: complete response, PR: partial response, S: stability, P: progression, BS: bone scan, FCH PET/CT: 18F-fluorocholine positron emission tomography/computed tomography.

**Table 6:** Association of clinical and imaging variables with therapeutic failure.

Variables	χ²	*p* Value
Clinical and disease characteristics before ^223^Ra		
Bone events before ^223^Ra (categorical)	1.03	0.31
Gleason	3.438	0.931
Gleason (categorical)	0.566	0.452
ECOG	6.188	0.038
Prostatectomy (radical prostate cancer treatment)	4.386	0.036
RT (radical prostate cancer treatment)	0.23	0.632
Enzalutamide (before ^223^Ra)	0.038	0.845
Abiraterone (before ^223^Ra)	0.167	0.683
Docetaxel (before ^223^Ra)	0.114	0.736
Second-line ChT (before ^223^Ra)	0.376	0.54
Xofigo line	3.797	0.62
PSA progression during ^223^Ra	19.378	<0.001
PSA progression during ^223^Ra detailed 1 (^i^)	5.145	0.023
PSA progression during ^223^Ra detailed 2 (^ii^)	18.564	<0.001
Baseline AP	4.735	0.03
Baseline LDH	4.247	0.039
Clinical and disease characteristics after ^223^Ra		
Bone events during or after ^223^Ra (categorical)	0.41	0.84
Bone protective treatment (categorical)	5.213	0.04
Death (during follow-up)	11.335	<0.001
Progression (during follow-up)	2.43	0.253
PFS (£6 months vs. >6 months)	23.522	<0.001
OS (£12 months vs. >12 months)	18.446	<0.001
Baseline imaging technique results		
BM location on BS	2.304	0.129
Number of BM on BS	3.3	0.069
Extent of BM on baseline BS	4.637	0.205
High tumor burden of BM on BS	1.674	0.196
BM location on FCH PET/CT	6.584	0.01
Number of BM on FCH PET/CT	10.321	0.001
Extent of BM on baseline FCH PET/CT	14.998	0.001
BM characteristics on CT portion of FCH PET/CT	7.299	0.025
BM characteristics on CT portion FCH PET/CT (categorical) (^iii^)	7.016	0.008
Soft tissue involvement on FCH PET/CT	4.141	0.042
Concordance BS/FCH PET/CT (^iv^)	1.729	0.421
A pattern (BS vs. FCH PET/CT)	3.972	0.146
B pattern (BS)	0.343	0.558
C pattern (FCH PET/CT)	3.434	0.064
D pattern (FCH PET/CT)	8.318	0.004

ChT: chemotherapy, RT: radiotherapy, BM: bone metastases, AP: alkaline phosphatase, LDH: lactate dehydrogenase, ECOG: Eastern Cooperative Oncology Group, PFS: progression-free survival, OS: overall survival, BS: bone scintigraphy, FCH PET/CT: 18F-fluorocholine positron emission tomography/computed tomography, CT: computed tomography, A pattern: osteogenic or metabolic activity predominance, B pattern: bone marrow involvement in bone scintigraphy, C pattern: bone marrow involvement on FCH PET, D pattern: PET uptake on BM higher than liver uptake, (^i^) first part vs. second part of ^223^Ra treatment, (^ii^) first part of ^223^Ra treatment vs. ulterior, (^iii^) osteoblastic vs. rest (mixed and osteolytic), (^iv^) concordance BS/PET: good, moderate, or bad.

**Table 7:** Association of clinical and imaging variables with skeletal events during or after ^223^Ra treatment.

Variables	χ²	*p* Value
Patient and clinical characteristics before ^223^Ra		
Bone events before ^223^Ra (yes/no)	1.198	0.274
Bone events before ^223^Ra (type)	8.473	0.029
Gleason	7.45	0.395
ECOG	2.422	0.375
^223^Ra line	3.624	0.651
PSA progression during ^223^Ra	0.498	0.48
PSA progression during ^223^Ra detailed 1	0.048	1
PSA progression during ^223^Ra detailed 2	0.256	0.613
Baseline AP	1.455	0.228
Baseline LDH	0.164	0.759
Clinical and disease characteristics after ^223^Ra		
Additional treatments (categorical)	2.157	0.225
Additional treatments (line number)	18.544	0.001
Additional treatment (Abiraterone)	3.835	0.05
Additional treatment (Enzalutamide)	1.127	0.288
Additional treatment (ChT)	5.963	0.058
Bone protective treatment (categorical)	0.092	1
Bone protective treatment (descriptive)	2.816	0.25
Bone protective treatment (moment)	1.117	0.771
Death during follow-up	0.851	0.449
Progression during follow-up	0.086	1
PFS (£6 months vs. >6 months)	0.837	0.36
OS (£12 months vs. >12 months)	7.787	0.005
Baseline imaging techniques		
BM location on BS	0.013	0.91
Number of BM on BS	0.217	0.641
Extent of BM on baseline BS	0.316	0.972
BM high tumor burden on BS	0	0.992
BM on FCH PET/CT	3.253	0.071
Number of BM on FCH PET/CT	0.205	0.651
Extent of BM on FCH PET/CT	1.27	0.752
BM high tumor burden on FCH PET/CT	5.256	0.022
BM characteristics on CT portion of FCH PET/CT (descriptive)	4.036	0.134
BM characteristics on CT portion of FCH PET/CT (categorical)	0.368	0.618
Soft tissue involvement on FCH PET/CT (categorical)	1.417	0.234
Soft tissue involvement of FCH PET/CT (descriptive)	1.273	0.718
Concordance BS/FCH PET/CT 1	0.842	0.622
Concordance BS/FCH PET/CT 2	0.031	1
A pattern (BS vs. FCH PET/CT)	1.674	0.457
B pattern (BS)	1.218	0.27
C pattern (FCH PET/CT)	5.088	0.033
D pattern (FCH PET/CT)	3.594	0.058
Interim imaging techniques		
Interim BS response	0.105	0.904
Interim BS response (categorical)	0.101	0.751
Interim FCH PET/CT response	0.115	1
Interim FCH PET/CT response (categorical)	0.028	0.867
Progression localization	2.856	0.463
Concordance BS/FCH PET/CT 1	0.523	0.768
Concordance BS/FCH PET/CT 2	0.651	0.522
End-treatment imaging techniques		
End-treatment BS response	3.288	0.213
End-treatment BS response	1.081	0.427
End-treatment FCH PET/CT response	3.934	0.287
End-treatment FCH PET/CT response	2.438	0.155
Progression location	2.526	0.906
Concordance BS/FCH PET/CT 1	0.891	0.646
Concordance BS/FCH PET/CT 2	0.107	1

ECOG: Eastern Cooperative Oncology Group, ChT: chemotherapy, RT: radiotherapy, BS: bone scan, FCH PET/CT: 18F-fluorocholine positron emission tomography/computed tomography, CT: portion of FCH PET/CT, BM: Bone metastases, BS: Bone scintigraphy, PSA progression during ^223^Ra detailed 1: progression at first or second 3 doses, PSA progression during ^223^Ra detailed 2: progression at 3 first doses to second 3 and beyond, PFS categorical: progression-free survival > 6 months or not, OS categorical: overall survival > 12 months or not, concordance BS/PET 1: good, moderate, or bad, concordance BS/FCH PET/CT 2: good/moderate or bad, A pattern: osteogenic or metabolic activity predominance, B pattern: bone marrow involvement in BS, C Pattern: bone marrow involvement on FCH PET, D pattern: FCH PET uptake higher than liver uptake.

**Table 8:** Results of the Cox regression index of clinical and imaging quantitative variables with overall survival.

Variables	HR	95% CI of HR	*p*-Value
Doses of ^223^Ra (number)	0.699	0.606–0.807	<0.001
Patient age	1.005	0.981–1.030	0.666
Time from diagnosis of pCa to ^223^Ra	0.122	0.995–1.003	0.727
Time of evolution of BM to ^223^Ra	0.999	0.993–1.007	0.925
Time of CRPC to ^223^Ra	1.003	0.994–1.012	0.479
Baseline PSA	1	1.0001–1.0004	0.028
Baseline AP	1.002	1.001–1.004	<0.001
Baseline LDH	1	0.999–1.001	0.511
SUVmax	1.049	1.006–1.092	0.023
Average SUVmax	1.07	1.012–1.132	0.017

HR: hazard ratio, CI: confidence interval, pCa: prostate cancer, CRPC: castration-resistant prostate cancer, BM: bone metastases, PSA: prostate-specific antigen, AP: alkaline phosphatase, LDH: lactate dehydrogenase, SUV: standardized uptake value.

**Table 9:** Log-rank (Mantel–Cox) results of association of clinical and tumor characteristics with overall survival.

Variables		Median (mo)	χ²	*p*-Value
Bone events before ^223^Ra	Yes	16	0.022	0.883
	No	13		
Bone events during or after ^223^Ra	Yes	19	4.998	0.025
	No	12		
Any treatments after ^223^Ra	Yes	14	25.699	<0.001
	No	5		
Abiraterone after ^223^Ra	Yes	23	6.614	0.01
	No	13		
Enzalutamide after ^223^Ra	Yes	23	11.312	<0.001
	No	13		
Bone protective treatment	Yes	14	0.113	0.737
	No	14		
Bone protective treatment (Denosumab)	Zolendronic	16	4.228	0.04
	Both	8	0.329	0.566
Bone protective treatment (Zolendronic)	Denosumab	13	4.228	0.04
	Both	8	1.506	0.22
Therapeutic failure	Yes	8	29.1	<0.001
	No	21		
Gleason (categorical)	≥8	14	0.036	0.85
	Rest	14		
ECOG 0	1	9	4.253	0.039
	2	5	23.337	<0.001
ECOG 1	2	5	3.874	0.049
Enzalutamide before ^223^Ra	Yes	10	1.081	0.299
	No	16		
Abiraterone before ^223^Ra	Yes	13	2.663	0.103
	No	18		
Second-line ChT before ^223^Ra	Yes	9	0.119	0.73
	No	14		
First Docetaxel before ^223^Ra	Yes	14	0.958	0.328
	No	14		
^223^Ra line 1	2	15	4.168	0.041
	3	11	0.851	0.356
	4	14	0.243	0.622
	5	7	5.279	0.022
	7	8	3	0.083
^223^Ra line 2	3	11	0.051	0.821
	4	14	0.563	0.453
	5	7	5.171	0.023
	7	8	3.725	0.054
^223^Ra line 3	4	14	0.766	0.381
	5	7	1.715	0.19
	7	8	0.551	0.458
^223^Ra line 4	5	7	3.208	0.073
	7	8	1.548	0.213
^223^Ra line 5	7	8	0.141	0.707
^223^Ra line (categorical 1)	First 3	14	0.015	0.902
	4th and beyond	11		
^223^Ra line (categorical 2)	First 2	19	0.36	0.549
	3rd and beyond	11		
PSA progression during ^223^Ra	Yes	12	8.508	0.004
	No	19		
PSA progression detailed 1	A	12	0.122	0.726
	B	11		
PSA progression detailed 2	A	12	5.693	0.017
	C	16		
Baseline AP	Pathological	8	12.581	<0.001
	Normal	15		
Baseline LDH	Pathological	8	4.526	0.033
	Normal	14		

PFS: progression-free survival, mo: months, χ^2^: chi-squared, ECOG: Eastern Cooperative Oncology Group, BS: bone scan, FCH PET/CT: 18F-fluorocholine positron emission tomography/computed tomography, PSA progression A: progression at first 3 doses, PSA progression B: progression at 3 s doses, PSA progression C: progression at 3 s doses and beyond.

**Table 10:** Log-rank (Mantel–Cox) results of association of baseline imaging variables with overall survival.

Baseline Imaging Variables		Median (mo)	χ²	*p*-Value
BM location on BS	Axial	15	1.414	0.234
	Axial and other	11		
BM number on BS	Oligometastatic	17	2.534	0.111
	Polimetastasic	11		
Number of BM on BS (1–5 lesions)	6–20 lesions	13	0.715	0.398
	>20 lesions	8	3.454	0.063
	Superscan	5	2.531	0.112
Number of BM on BS (6–20 lesions)	>20 lesions	8	1.289	0.256
	Superscan	5	0.703	0.402
Number of BM on BS (>20 lesions)	Superscan	5	0.117	0.732
High burden of BM on BS	Yes	11	1.691	0.193
	No	16		
BM location on FCH PET/CT	Axial	21	14.769	<0.001
	Axial and other	8		
Number of BM on FCH PET/CT	Oligometastatic	18	4.814	0.028
	Polimetastasic	10		
Number of BM on FCH PET/CT (1–5)	6–20	13	1.215	0.27
	>20	8	7.259	0.007
	Superscan	5	16.751	<0.001
Number of BM on FCH PET/CT (6–20)	>20	8	3.577	0.059
	Superscan	5	9.494	0.002
Number of BM on FCH PET/CT (>20)	Superscan	5	2.081	0.149
FCH PET/CT high tumor burden of BM	Yes	9	9.016	0.003
	No	18		
BM characteristics (^i^) on CT (osteoblastic)	Osteolytic	13	0.196	0.658
	Mixed	9	3.944	0.047
BM characteristics on CT (osteolytic)	Mixed	9	0.953	0.329
BM characteristics on CT (categorical)	Osteoblastic	14	2.62	0.106
	Rest	12		
Soft tissue involvement on FCH PET/CT	Yes	7	3.911	0.048
	No	14		
BS/PET concordance (good)	Moderate	8	4.988	0.026
	Bad	13	0.896	0.344
BS/PET concordance (moderate)	Bad	13	5.211	0.022
A pattern	Osteogenic activity predominance	15	3.718	0.054
	Similar uptake	7	0.193	0.66
A pattern	Similar uptake	7	0.879	0.348
B pattern	Yes	7	2.626	0.105
	No	14		
C pattern	Yes	5	27.266	<0.001
	No	14		
D pattern	Higher than liver	9	13.17	<0.001
	Lower than liver	18		

χ²: chi-squared, mo: months, BM: bone metastases, BS: bone scan, FCH PET/CT: 18F-fluorocholine positron emission tomography/computed tomography, CT: portion of FCH PET/CT, A pattern: osteogenic or metabolic activity predominance, B pattern: bone marrow involvement in bone scintigraphy, C pattern: bone marrow involvement on FCH PET, D pattern: PET uptake higher than liver uptake, (^i^) on CT portion of FCH PET/CT.

**Table 11:** Log-rank (Mantel–Cox) results of association of treatment response imaging variables with overall survival.

Variables		Median (mo)	χ²	*p*-Value
Interim imaging techniques				
BS interim response (partial response, 12 mo)	Stability	17	0.018	0.892
	Progression	13	0.836	0.361
BS interim response (stability)	Progression	13	8.723	0.003
BS interim response categorical	No progression	15	8.371	0.004
	Progression	13		
Interim PET response (partial response, 21 mo)	Stability	23	0.011	0.917
	Progression	11	7.044	0.008
Interim PET response (stability)	Progression	11	13.759	<0.001
Interim PET response categorical	No progression	23	17.026	<0.001
	Progression	11		
Interim PET progression (bone, 8 mo)	Adenopathic	14	1.543	0.214
	Bone and adenopathic	14	0.42	0.517
	Bone and visceral	9	0.066	0.797
Interim PET progression (adenopathic)	Bone and adenopathic	14	0.347	0.556
	Bone and visceral	9	0.656	0.418
Interim PET progression (bone and adenopathic)	Bone and visceral	9	1.101	0.294
End-treatment images techniques				
End-treatment BS response (partial response 23 mo)	Stability	21	0.005	0.946
	Progression	16	2.598	0.107
End-treatment BS response (stability)	Progression	16	2.944	0.086
End-treatment BS response categorical	No progression	23	3.47	0.063
	Progression	16		
End-treatment PET response (complete response, 24 mo)	Partial response	34	0.059	0.808
	Stability	17	0.128	0.72
	Progression	19	0.553	0.457
End-treatment PET response (partial response)	Stability	17	0.089	0.765
	Progression	19	0.373	0.542
End-treatment PET response (stability)	Progression	19	0.264	0.607
End-treatment PET response categorical	No progression	23	1.327	0.249
	Progression	18		
End-treatment PET progression (bone, 15 mo)	Adenopathic	18	0.022	0.883
	Visceral	10	2.762	0.097
	Bone and adenopathic	19	1.185	0.276
	Bone and visceral	47	2.166	0.141
End-treatment PET progression (adenopathic)	Visceral	10	3	0.083
	Bone and adenopathic	19	1.182	0.277
	Bone and visceral	47	1.779	0.182
End-treatment PET progression (visceral)	Bone and adenopathic	19	2	0.157
	Bone and visceral	47	1	0.317
End-treatment PET progression (bone and adenopathic)	Bone and visceral	47	1.471	0.225

χ²: chi-squared, mo: months, BM: bone metastases, BS: bone scan, FCH PET/CT: 18F-fluorocholine positron emission tomography/computed tomography, CT: portion of FCH PET/CT.

## Data Availability

Data is unavailable due to privacy or ethical restrictions.

## References

[1] Parker C., Castro E., Fizazi K., Heidenreich A., Ost P., Procopio G., Tombal B., Gillessen S. (2020). ESMO Guidelines Committee. Prostate Cancer: ESMO Clinical Practice Guidelines for Diagnosis, Treatment and Follow-Up. Ann. Oncol..

[2] Mottet N., van den Bergh R.C.N., Briers E., den Broeck T.V., Cumberbatch M.G., de Santis M., Fanti S., Fossati N., Gandaglia G., Gillessen S. (2021). EAU-EANM-ESTRO-ESUR-SIOG Guidelines on Prostate Cancer-2020 Update. Part 1: Screening, Diagnosis, and Local Treatment with Curative Intent. Eur Urol..

[3] Schaeffer E.M., Srinivas S., Adra N., An Y., Barocas D., Bitting R., Bryce A., Chapin B., Cheng H.H., D’Amico A.V. (2023). Prostate Cancer, Version 4.2023, NCCN Clinical Practice Guidelines in Oncology. J. Natl. Compr. Canc. Netw..

[4] Lowrance W., Dreicer R., Jarrard D.F., Scarpato K.R., Kim S.K., Kirkby E., Buckley D.I., Griffin J.C., Cookson M.S. (2023). Updates to Advanced Prostate Cancer: AUA/SUO Guideline (2023). J. Urol..

[5] Parker C., Nilsson S., Heinrich D., Helle S.I., O’Sullivan J.M., Fossa S.D., Chodacki A., Wiechno P., Logue J., Seke M. (2013). Alpha emitter radium-223 and survival in metastatic prostate cancer. N. Engl. J. Med..

[6] Smith M., Parker C., Saad F., Miller K., Tombal B., Ng Q.S., Boegemann M., Matveev V., Piulats J.M., Zucca L.E. (2019). Addition of radium-223 to abiraterone acetate and prednisone or prednisolone in patients with castration-resistant prostate cancer and bone metastases (ERA 223): A randomised, double-blind, placebo-controlled, phase 3 trial. Lancet Oncol..

[7] Brady D., Parker C.C., O’Sullivan J.M. (2013). Bone-targeting radiopharmaceuticals including radium-223. Cancer J..

[8] Fuccio C., Castellucci P., Schiavina R., Guidalotti P.L., Gavaruzzi G., Montini G.C., Nanni C., Marzola M.C., Rubello D., Fanti S. (2012). Role of 11C-choline PET/CT in the restaging of prostate cancer patients with biochemical relapse and negative results at bone scintigraphy. Eur. J. Radiol..

[9] Beheshti M., Vali R., Waldenberger P., Fitz F., Nader M., Loidl W., Broinger G., Stoiber F., Foglman I., Langsteger W. (2008). Detection of bone metastases in patients with prostate cancer by 18F fluorocholine and 18F fluoride PET-CT: A comparative study. Eur. J. Nucl. Med. Mol. Imaging.

[10] García Vicente A.M., Amo-Salas M., Cassinello Espinosa J., Gómez Díaz R., Soriano Castrejón Á. (2021). Interim and end-treatment 18F-Fluorocholine PET/CT and bone scan in prostate cancer patients treated with Radium 223 dichloride. Sci. Rep..

[11] García Vicente A.M., González García B., Amo-Salas M., García Carbonero I., Cassinello Espinosa J., Gómez-Aldaraví Gutierrez J.L., Suarez Hinojosa L., Soriano Castrejón Á. (2019). Baseline 18F-Fluorocholine PET/CT and bone scan in the outcome prediction of patients treated with radium 223 dichloride. Clin. Transl. Oncol..

[12] Sartor O., Coleman R.E., Nilsson S., Heinrich D., Helle S.I., O’Sullivan J.M., Vogelzang N.J., Bruland O., Kobina S., Wilhelm S. (2017). An exploratory analysis of alkaline phosphatase, lactate dehydrogenase, and prostate-specific antigen dynamics in the phase 3 ALSYMPCA trial with radium-223. Ann. Oncol..

[13] Heinrich D., Bruland O., Guise T.A., Suzuki H., Sartor O. (2018). Alkaline phosphatase in metastatic castration-resistant prostate cancer: Reassessment of an older biomarker. Future Oncol..

[14] Alva A., Nordquist L., Daignault S., George S., Ramos J., Albany C., Isharwal S., McDonald M., Campbell G., Danchaivijitr P. (2017). Clinical Correlates of Benefit from Radium-223 Therapy in Metastatic Castration Resistant Prostate Cancer. Prostate.

[15] Fosbøl M.Ø., Petersen P.M., Kjaer A., Mortensen J. (2018). ^223^Ra Therapy of Advanced Metastatic Castration-Resistant Prostate Cancer: Quantitative Assessment of Skeletal Tumor Burden for Prognostication of Clinical Outcome and Hematologic Toxicity. J. Nucl. Med..

[16] Kitajima K., Igeta M., Kuyama J., Kawahara T., Suga T., Otani T., Sugawara S., Kono Y., Tamaki Y., Seko-Nitta A. (2023). Novel nomogram developed for determining suitability of metastatic castration-resistant prostate cancer patients to receive maximum benefit from radium-223 dichloride treatment-Japanese Ra-223 Therapy in Prostate Cancer using Bone Scan Index (J-RAP-BSI) Trial. Eur. J. Nucl. Med. Mol. Imaging.

[17] Shariftabrizi A., Kothari S., George S., Attwood K., Levine E., Lamonica D. (2023). Optimization of Radium-223 Treatment for Castration-resistant Prostate Cancer: Insights from Skeletal Metastasis Burden and Clinical Parameters. Cancers.

[18] Crawford E.D., Petrylak D.P., Shore N., Saad F., Slovin S.F., Vogelzang N.J., Keane T.E., Koo P.J., Gomella L.G., O’Sullivan J.M. (2017). Prostate Cancer Radiographic Assessments for Detection of Advanced Recurrence (RADAR II) Group. The Role of Therapeutic Layering in Optimizing Treatment for Patients with Castration-resistant Prostate Cancer (Prostate Cancer Radiographic Assessments for Detection of Advanced Recurrence II). Urology.

[19] Slack N.H., Karr J.P., Chu T.M., Murphy G.P. (1980). An assessment of bone scans for monitoring osseous metastases in patients being treated for prostate carcinoma. Prostate.

[20] Young H., Baum R., Cremerius U., Herholz K., Hoekstra O., Lammertsma A.A., Pruim J., Price P. (1999). Measurement of clinical and subclinical tumour response using [18F]-fluorodeoxyglucose and positron emission tomography: Review and 1999 EORTC recommendations. European Organization for Research and Treatment of Cancer (EORTC) PET Study Group. Eur. J. Cancer.

[21] European Medicines Agency (EMA) (2020). Provisional Assessment Report: Provisional Measures under Article 20—XOFIGO (H-20-1459-C-002653-0028). https://www.ema.europa.eu/en/documents/variation-report/xofigo-h-20-1459-c-002653-0028-epar-assessment-report-provisional-measures-article-20_en.pdf.

[22] Parikh S., Murray L., Kenning L., Bottomley D., Din O., Dixit S., Ferguson C., Handforth C., Joseph L., Mokhtar D. (2018). Real-world Outcomes and Factors Predicting Survival and Completion of Radium 223 in Metastatic Castrate-resistant Prostate Cancer. Clin. Oncol..

[23] Wong W.W., Anderson E.M., Mohammadi H., Daniels T.B., Schild S.E., Keole S.R., Choo C.R., Tzou K.S., Bryce A.H., Ho T.H. (2017). Factors Associated with Survival Following Radium-223 Treatment for Metastatic Castration-resistant Prostate Cancer. Clin. Genitourin. Cancer.

[24] Kuppen M.C., Westgeest H.M., van der Doelen M.J., van den Eertwegh A.J., Coenen J.L., Aben K.K., van den Bergh A.C., Bergman A.M., den Bosch J.V., Celik F. (2020). Real-world outcomes of radium-223 dichloride for metastatic castration resistant prostate cancer. Future Oncol..

[25] Frantellizzi V., Farcomeni A., Follacchio G.A., Pacilio M., Pellegrini R., Pani R., De Vincentis G. (2018). A 3-variable prognostic score (3-PS) for overall survival prediction in metastatic castration-resistant prostate cancer treated with ^223^Radium-dichloride. Ann. Nucl. Med..

[26] Bauckneht M., Rebuzzi S.E., Ponzano M., Borea R., Signori A., Frantellizzi V., Lodi Rizzini E., Mascia M., Lavelli V., Miceli A. (2022). Prognostic Value of the BIO-Ra Score in Metastatic Castration-Resistant Prostate Cancer Patients Treated with Radium-223 after the European Medicines Agency Restricted Use: Secondary Investigations of the Multicentric BIO-Ra Study. Cancers.

[27] van der Doelen M.J., Oving I.M., Wyndaele D.N.J., van Basten J.P., Terheggen F., van de Luijtgaarden A.C.M., Oyen W.J.G., van Schelven W.D., van den Berkmortel F., Mehra N. (2023). Health-Related Quality of Life, Psychological Distress, and Fatigue in Metastatic Castration-Resistant Prostate Cancer Patients Treated with Radium-223 Therapy. Prostate Cancer Prostatic Dis..

[28] Ge R., Wang Z., Cheng L. (2022). Tumor microenvironment heterogeneity an important mediator of prostate cancer progression and therapeutic resistance. NPJ Precis. Oncol..

[29] Tolkach Y., Kristiansen G. (2018). The Heterogeneity of Prostate Cancer: A Practical Approach. Pathobiology.

[30] Yamamoto Y., Okuda Y., Kanaki T., Tanaka R., Nagahara A., Nakai Y., Nakayama M., Kakimoto K.I., Nishimura K. (2021). Clinical indicators for predicting prognosis after radium-223 administration in castration-resistant prostate cancer with bone metastases. Int. J. Clin. Oncol..

[31] Palmedo H., Ahmadzadehfar H., Eschmann S., Niesen A., Schönberger J., Barsegian V., Liepe K., Mottaghy F.M., Guan R., Pinkert J. (2023). Pain Outcomes in Patients with Metastatic Castration-Resistant Prostate Cancer Treated with ^223^Ra: PARABO, a Prospective, Noninterventional Study. J. Nucl. Med..

[32] Buonerba C., Ferro M., Dolce P., Crocetto F., Verde A., Lucarelli G., Scafuri L., Facchini S., Vaia A., Marinelli A. (2020). Predictors of efficacy of androgen-receptor-axis-targeted therapies in patients with metastatic castration-sensitive prostate cancer: A systematic review and meta-analysis. Crit. Rev. Oncol./Hematol..

[33] Saad F., Carles J., Gillessen S., Heidenreich A., Heinrich D., Gratt J., Lévy J., Miller K., Nilsson S., Petrenciuc O. (2016). Radium-223 International Early Access Program Investigators. Radium-223 and concomitant therapies in patients with metastatic castration-resistant prostate cancer: An international, early access, open-label, single-arm phase 3b trial. Lancet Oncol..

[34] Ramos J.D., Mostaghel E.A., Pritchard C.C., Yu E.Y. (2018). DNA Repair Pathway Alterations in Metastatic Castration-resistant Prostate Cancer Responders to Radium-223. Clin. Genitourin. Cancer.

[35] Steinberger A.E., Cotogno P., Ledet E.M., Lewis B., Sartor O. (2017). Exceptional Duration of Radium-223 in Prostate Cancer with a BRCA2 Mutation. Clin. Genitourin. Cancer.

[36] Armstrong A.J., Anand A., Edenbrandt L., Bondesson E., Bjartell A., Widmark A., Sternberg C.N., Pili R., Tuvesson H., Nordle Ö. (2018). Phase 3 Assessment of the Automated Bone Scan Index as a Prognostic Imaging Biomarker of Overall Survival in Men with Metastatic Castration-Resistant Prostate Cancer: A Secondary Analysis of a Randomized Clinical Trial. JAMA Oncol..

[37] van der Zande K., Oyen W.J.G., Zwart W., Bergman A.M. (2021). Radium-223 Treatment of Patients with Metastatic Castration Resistant Prostate Cancer: Biomarkers for Stratification and Response Evaluation. Cancers.

[38] de Jong A.C., Segbers M., Ling S.W., Graven L.H., Mehra N., Hamberg P., Brabander T., de Wit R., van der Veldt A.A.M. (2023). 68Ga-PSMA PET/CT for Response Evaluation of ^223^Ra Treatment in Metastatic Prostate Cancer. J. Nucl. Med..

[39] Donners R., Tunariu N., Tovey H., Hall E., Chua S., Cook G., Du Y., Blackledge M.D., Parker C.C., Koh D.M. (2024). The value of baseline 18F-sodium fluoride and 18F-choline PET activity for identifying responders to radium-223 treatment in castration-resistant prostate cancer bone metastases. Eur. Radiol..

[40] Fuccio C., Castellucci P., Schiavina R., Santi I., Allegri V., Pettinato V., Boschi S., Martorana G., Al-Nahhas A., Rubello D. (2010). Role of 11C-choline PET/CT in the restaging of prostate cancer patients showing a single lesion on bone scintigraphy. Ann. Nucl. Med..

[41] Bosch D., van der Velden K.J.M., Oving I.M., Wyndaele D.N.J., Weijs L.E., van Schelven W.D., Oyen W.J.G., Te Beek E.T., van de Luijtgaarden A.C.M., Somford D.M. (2024). The Impact of Baseline PSMA PET/CT Versus CT on Outcomes of ^223^Ra Therapy in Metastatic Castration-Resistant Prostate Cancer Patients. J. Nucl. Med..

[42] Filippi L., Spinelli G.P., Chiaravalloti A., Schillaci O., Equitani F., Bagni O. (2020). Prognostic Value of 18F-Choline PET/CT in Patients with Metastatic Castration-Resistant Prostate Cancer Treated with Radium-223. Biomedicines.

[43] Maines F., Caffo O., Donner D., Sperduti I., Bria E., Veccia A., Chierichetti F., Tortora G., Galligioni E. (2016). Serial 18F-choline-PET Imaging in Patients Receiving Enzalutamide for Metastatic Castration-Resistant Prostate Cancer: Response Assessment and Imaging Biomarkers. Future Oncol..

[44] Caroli P., De Giorgi U., Scarpi E., Fantini L., Moretti A., Galassi R., Celli M., Conteduca V., Rossi L., Bianchi E. (2018). Prognostic value of 18F-choline PET/CT metabolic parameters in patients with metastatic castration-resistant prostate cancer treated with abiraterone or enzalutamide. Eur. J. Nucl. Med. Mol. Imaging.

[45] Vija Racaru L., Sinigaglia M., Kanoun S., Ben Bouallègue F., Tal I., Brillouet S., Bauriaud-Mallet M., Zerdoud S., Dierickx L., Vallot D. (2018). Fluorine-18-fluorocholine PET/CT parameters predictive for hematological toxicity to radium-223 therapy in castrate-resistant prostate cancer patients with bone metastases: A pilot study. Nucl. Med. Commun..

[46] Quaquarini E., D’Ambrosio D., Sottotetti F., Gallivanone F., Hodolic M., Baiardi P., Palumbo R., Vellani C., Canevari C., Bernardo A. (2019). Prognostic Value of 18 F-Fluorocholine PET Parameters in Metastatic Castrate-Resistant Prostate Cancer Patients Treated with Docetaxel. Contrast Media Mol. Imaging.

[47] Letellier A., Johnson A.C., Kit N.H., Savigny J.F., Batalla A., Parienti J.J., Aide N. (2018). Uptake of Radium-223 Dichloride and Early [18F]NaF PET Response Are Driven by Baseline [18F]NaF Parameters: A Pilot Study in Castration-Resistant Prostate Cancer Patients. Mol. Imaging Biol..

[48] Etchebehere E.C., Araujo J.C., Fox P.S., Swanston N.M., Macapinlac H.A., Rohren E.M. (2015). Prognostic factors in patients treated with 223Ra: The role of skeletal tumor burden on baseline 18F-fluoride PET/CT in predicting overall survival. J. Nucl. Med..

[49] Ahmadzadehfar H., Azgomi K., Hauser S., Wei X., Yordanova A., Gaertner F.C., Kürpig S., Strunk H., Essler M. (2017). 68Ga-PSMA-11 PET as a Gatekeeper for the Treatment of Metastatic Prostate Cancer with ^223^Ra: Proof of Concept. J. Nucl. Med..

[50] Anand A., Trägårdh E., Edenbrandt L., Beckman L., Svensson J.H., Thellenberg C., Widmark A., Kindblom J., Ullén A., Bjartell A. (2020). Assessing Radiographic Response to ^223^Ra with an Automated Bone Scan Index in Metastatic Castration-Resistant Prostate Cancer Patients. J. Nucl. Med..

[51] van der Doelen M.J., Stockhaus A., Ma Y., Mehra N., Yachnin J., Gerritsen W.R., Nilsson S., van Oort I.M., Ullén A. (2021). Early alkaline phosphatase dynamics as biomarker of survival in metastatic castration-resistant prostate cancer patients treated with radium-223. Eur. J. Nucl. Med. Mol. Imaging.

[52] Gafita A., Rauscher I., Weber M., Hadaschik B., Wang H., Armstrong W.R., Tauber R., Grogan T.R., Czernin J., Rettig M.B. (2022). Novel Framework for Treatment Response Evaluation Using PSMA PET/CT in Patients with Metastatic Castration-Resistant Prostate Cancer (RECIP 1.0): An International Multicenter Study. J. Nucl. Med..

[53] Fanti S., Hadaschik B., Herrmann K. (2020). Proposal for Systemic-Therapy Response-Assessment Criteria at the Time of PSMA PET/CT Imaging: The PSMA PET Progression Criteria. J. Nucl. Med..

[54] Fanti S., Goffin K., Hadaschik B.A., Herrmann K., Maurer T., MacLennan S., Oprea-Lager D.E., Oyen W.J., Rouvière O., Mottet N. (2021). Consensus Statements on PSMA PET/CT Response Assessment Criteria in Prostate Cancer. Eur. J. Nucl. Med. Mol. Imaging.

[55] Lange P.H., Vessella R.L. (1998). Mechanisms, hypotheses and questions regarding prostate cancer micrometastases to bone. Cancer Metastasis Rev..

[56] Parker C.C., Coleman R.E., Sartor O., Vogelzang N.J., Bottomley D., Heinrich D., Helle S.I., O’Sullivan J.M., Fossà S.D., Chodacki A. (2018). Three-year Safety of Radium-223 Dichloride in Patients with Castration-resistant Prostate Cancer and Symptomatic Bone Metastases from Phase 3 Randomized Alpharadin in Symptomatic Prostate Cancer Trial. Eur. Urol..

[57] Sartor O., Coleman R., Nilsson S., Heinrich D., Helle S.I., O’Sullivan J.M., Fosså S.D., Chodacki A., Wiechno P., Logue J. (2014). Effect of radium-223 dichloride on symptomatic skeletal events in patients with castration-resistant prostate cancer and bone metastases: Results from a phase 3, double-blind, randomised trial. Lancet Oncol..

[58] Kitajima K., Kuyama J., Kawahara T., Suga T., Otani T., Sugawara S., Kono Y., Tamaki Y., Seko-Nitta A., Ishiwata Y. (2023). Assessing Therapeutic Response to Radium-223 with an Automated Bone Scan Index among Metastatic Castration-Resistant Prostate Cancer Patients: Data from Patients in the J-RAP-BSI Trial. Cancers.

[59] van der Doelen M.J., Kuppen M.C.P., Jonker M.A., Mehra N., Janssen M.J.R., van Oort I.M., Gerritsen W.R. (2018). ^223^Ra Therapy in Patients with Advanced Castration-Resistant Prostate Cancer with Bone Metastases: Lessons from Daily Practice. Clin. Nucl. Med..

[60] McKay R.R., Jacobus S., Fiorillo M., Ledet E.M., Cotogna P.M., Steinberger A.E., Jacene H.A., Sartor O., Taplin M.E. (2017). Radium-223 Use in Clinical Practice and Variables Associated with Completion of Therapy. Clin. Genitourin. Cancer.

[61] Hashimoto K., Miyoshi Y., Shindo T., Hori M., Tsuboi Y., Kobayashi K., Fukuta F., Tanaka T., Miyamoto S., Maehana T. (2020). Dynamic Changes of Bone Metastasis Predict Bone-Predominant Status to Benefit from Radium-223 Dichloride for Patients with Castration-Resistant Prostate Cancer. Cancer Med..

[62] Miyoshi Y., Tsutsumi S., Yasui M., Kawahara T., Uemura K.I., Hayashi N., Nozawa M., Yoshimura K., Uemura H., Uemura H. (2021). A Novel Prediction Model. for the Completion of Six Cycles of Radium-223 Treatment and Survival in Patients with Metastatic Castration-Resistant Prostate Cancer. World J. Urol..

[63] Frantellizzi V., Pani A., Ippoliti M.D., Farcomeni A., Aloise I., Colosi M., Polito C., Pani R., Vincentis G. (2019). Scintigraphic Load of Bone Disease Evaluated by DASciS Software as a Survival Predictor in Metastatic Castration-Resistant Prostate Cancer Patients Candidates to ^223^RaCl Treatment. Radiol. Oncol..

[64] Nakashima K., Makino T., Kadomoto S., Iwamoto H., Yaegashi H., Iijima M., Kawaguchi S., Nohara T., Shigehara K., Izumi K. (2019). Initial Experience with Radium-223 Chloride Treatment at the Kanazawa University Hospital. Anticancer Res..

[65] Frantellizzi V., Monari F., Mascia M., Costa R., Rubini G., Spanu A., Di Rocco A., Lodi Rizzini E., Cindolo L., Licari M. (2020). Validation of the 3-Variable Prognostic Score (3-PS) in mCRPC Patients Treated with ^223^Radium-Dichloride: A National Multicenter Study. Ann. Nucl. Med..

[66] Buscombe J., Gillett D., Bird N., Powell A., Heard S., Aloj L. (2020). Quantifying the Survival Benefit of Completing All Six Cycles of Radium-223 Therapy in Patients with Castrate-Resistant Prostate Cancer with Predominant Bone Metastases. World J. Nucl. Med..

[67] Dadhania S., Alonzi R., Douglas S., Gogbashian A., Hughes R., Dalili D., Vasdev N., Adshead J., Lane T., Westbury C. (2018). Single-centre experience of use of radium 223 with clinical outcomes based on number of cycles and bone marrow toxicity. Anticancer Res..

[68] Uemura H., Uemura H., Nagamori S., Wakumoto Y., Kimura G., Kikukawa H., Yokomizo A., Mizokami A., Kosaka T., Masumori N. (2019). Three-year follow-up of a phase II study of radium-223 dichloride in Japanese patients with symptomatic castration-resistant prostate cancer and bone metastases. Int. J. Clin. Oncol..

[69] Sasaki D., Hatakeyama S., Kawaguchi H., Hatayama Y., Ishibashi Y., Kusaka A., Noro D., Tanaka T., Ito H., Okuyama Y. (2022). Effects of six-cycle completion and earlier use of radium-223 therapy on prognosis for metastatic castration-resistant prostate cancer: A real-world multicenter retrospective study. Urol. Oncol. Semin. Orig. Investig..

[70] Cheng S., Arciero V., Goldberg H., Tajzler C., Manganaro A., Kozlowski N., Rowbottom L., McDonald R., Chow R., Vasisht G. (2019). Population-based analysis of the use of radium-223 for bone-metastatic castration-resistant prostate cancer in Ontario, and of factors associated with treatment completion and outcome. Cancer Manag. Res..

[71] Hurwitz M., Buscombe J.R., Jacene H.A., Klitzke A.K., Lamonica D., Lu Y., Pryma D.A., Rohren E.M., Speer T.W., Subramaniam R.M. (2020). ACR-ACNM-ASTRO-SNMMI practice parameter for the performance of therapy with radium-223. Am. J. Clin. Oncol..

[72] Wymenga L.F., Boomsma J.H., Groenier K., Piers D.A., Mensink H.J. (2001). Routine bone scans in patients with prostate cancer related to serum prostate-specific antigen and alkaline phosphatase. BJU Int..

[73] Ebrahim T., Hadebe B., Aldous C., Tinarwo P., Nyakale N. (2022). Segmented linear correlations between bone scan index and prostate cancer biomarkers, alkaline phosphatase, and prostate specific antigen in patients with a Gleason score ≥7. Medicine.

